# The complete chloroplast genome of *Chrysanthemum zawadskii* Herbich (Asteraceae) isolated in Korea

**DOI:** 10.1080/23802359.2021.1934148

**Published:** 2021-06-14

**Authors:** Jinwook Baek, Suhyeon Park, Junho Lee, Juhyeon Min, Jongsun Park, Gun Woong Lee

**Affiliations:** a Jeonju AgroBio-Materials Institute, Jeonju-si, Republic of Korea; b InfoBoss Inc., Seoul, Republic of Korea; c InfoBoss Research Center, Seoul, Republic of Korea; d FromBio Co. Ltd, Suwon-si, Republic of Korea

**Keywords:** Chloroplast genome, *Chrysanthemum zawadskii*, Asteraceae, intraspecific variations, Korea

## Abstract

We have determined the complete chloroplast genome of *Chrysanthemum zawadskii* Herbich isolated in Korea. The circular chloroplast genome of *C. zawadskii* is 151,137 bp long and has four subregions: 83,041 bp of large single copy and 18,350 bp of small single copy regions are separated by 24,873 bp of inverted repeat regions including 133 genes (87 protein-coding genes, eight rRNA genes, 37 tRNAs, and one pseudogene). There are 65 to 152 single nucleotide polymorphisms and 33 to 64 insertion and deletion regions (178 bp to 372 bp in length) identified against three available chloroplast genomes of *C. zawadskii*. The phylogenetic tree shows that *C. zawadskii* is clustered as a paraphyletic group with *C. zawadskii* subsp. *coreanum*, displaying incongruency between species and clades.

*Chrysanthemum zawadskii* Herbich (Asteraceae: Asteroideae), is a native plant in Korea (Park et al. 2020) and has economic values as traditional medicinal resources (Shin et al. 2010) and ornamental plants (Kim et al. 2014). There was controversy over its scientific name; *Chrysanthemum* L. and *Dendranthema* (DC.) Des Moul. were treated as two independent genera for a while due to morphological diversity (Bremer and Humphries 1993; Bremer 1994). However, *Dendranthema* was finally treated as a synonym of *Chrysanthemum* by a decision of the International Botanical Congress in 1999 (Trehane 1995; Nicolson 1999). *Chrysanthemum* including *C. zawadskii* has considerable variations in morphology and ploidy within species and is still unresolved based on a few chloroplast and nuclear markers (Liu et al. 2012). We completed *C. zawadskii* chloroplast genome to understand the phylogenetic position of *C. zawadskii* based on multiple complete chloroplast genomes.

We sequenced DNA extracted from fresh leaves of *C. zawadskii* (N35°34′10″, E127°1′18″; Jeongeup-si, Jeollabuk-do, Korea; InfoBoss Cyber Herbarium (IN); IB-01081; Contact: Suhyeon Park, shpark817@infoboss.co.kr) using DNeasy Plant Mini Kit (QIAGEN, Hilden, Germany). Raw sequences obtained from Illumina NovaSeq6000 (Macrogen Inc., Korea) were filtered by Trimmomatic v0.33 (Bolger et al. 2014) and *de novo* assembled by Velvet v1.2.10 (Zerbino and Birney 2008). Gaps were closed with GapCloser v1.12 (Zhao et al. 2011), BWA v0.7.17 (Li et al. 2009), and SAMtools v1.9 (Li 2013) under the Genome Information System (GeIS™; http://geis.infoboss.co.kr/). Geneious Prime^®^ 2020.2.4 (Biomatters Ltd., Auckland, New Zealand) was used to annotate chloroplast genome based on *C. indicum* chloroplast (NC_020320.1; Xia et al. 2016).

*C. zawadskii* chloroplast genome (MW539687) is 151,137 bp (GC ratio is 37.5%) with four subregions: 83,041 bp of large single-copy (35.5%), 18,350 bp of small single-copy (SSC; 30.8%) regions, and 24,873 bp of a pair of inverted repeats (IR; 43.1%). It contains 133 genes (87 protein-coding genes, eight rRNAs, 37 tRNAs, and one *ycf1* pseudogene in an IR region); 18 genes (seven protein-coding genes, four rRNAs, and seven tRNAs) are duplicated in IR regions.

In comparison to Chinese *C. zawadskii* chloroplast (MG799556), 110 single nucleotide polymorphisms (SNPs) and 45 insertion and deletion (INDEL) regions (251 bp in length) were identified. Interestingly, 28 of 45 SNPs in 20 PCGs (62.2%) are non-synonymous SNPs (nsSNPs), similar to *Chenopodium album* (Park et al. 2021), suggesting that these variations can be used for developing molecular markers. Numbers of intraspecific variations of *C. zawadskii* are fewer than those identified between Korea and China samples (Heo, Kim, et al. 2019; Heo et al. 2020; Oh and Park 2020; Park et al. 2020), suggesting weak effects of geographical distribution. Additionally, numbers of intraspecific variations between our chloroplast and those of two varieties are 65 SNPs and 33 INDEL regions (178 bp in length) and 152 SNPs and 64 INDEL regions (372 bp in length), and 110 SNPs and 45 INDEL regions (251 bp in length), respectively. These variations are greater than those identified among different varieties, including *Potentilla freyniana* (Heo, Park, et al. 2019; Park, Heo, et al. 2019) and *Aconitum barbatum* (Chen et al. 2015), supporting that paraphyletic manner of *C. zawadskii* (Figure 1) together with larger amount of intraspecific variations than those identified from the Korean samples (Kim et al. 2019; Min et al. 2019; Park, Heo, et al. 2019; Park, Kim, and Xi 2019; Park, Kim, Xi, Oh, et al. 2019a, 2019b; Kim et al. 2020; Park and Oh 2020; Park et al. 2021).

**Figure 1.**
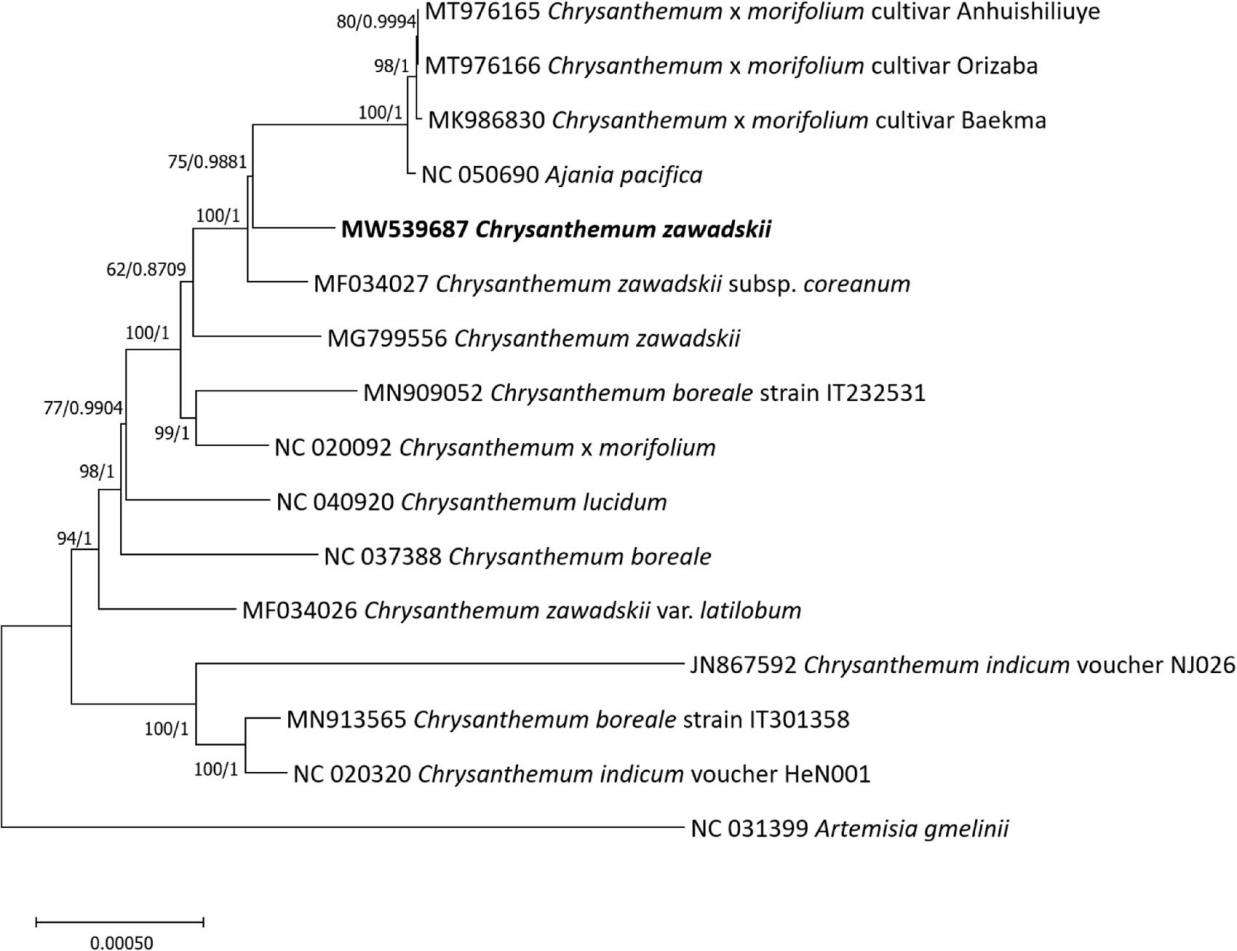
Maximum-Likelihood phylogenetic tree and Bayesian inference tree were constructed based on sixteen Asteraceae chloroplast genomes. Phylogenetic tree was drawn based on maximum-Likelihood phylogenetic tree. Values above branches are bootstrap supports from the analysis of Maximum-Likelihood and Bayesian posterior probabilities.

Sixteen Asteraceae complete chloroplast genomes including four *C. zawadskii* chloroplast genomes were aligned by MAFFT v7.450 (Katoh and Standley 2013) for constructing Maximum-Likelihood (ML) and Bayesian inference (BI) phylogenetic trees after adjusting SSC direction. A heuristic search was used with nearest-neighbor interchange branch swapping, the Tamura-Nei model, and uniform rates among sites to construct ML tree with 1,000 pseudo-replicates bootstrap option and default values of other options using MEGA X (Kumar et al. 2018). BI tree was constructed by MrBayes v3.2.6. (Ronquist et al. 2012). The GTR model with gamma rates was used. A Markov-chain Monte Carlo algorithm was employed for 1,100,000 generations, sampling trees every 200 generations, with four chains running simultaneously. Both trees show that two *C. zawadskii* chloroplast genomes are clustered with *C. zawadski*i subsp. *coreanum* and four *Chrysanthemum* chloroplast genomes by high supportive values (Figure 1). Moreover, phylogenetic trees display three incongruencies: i) *C. zawadskii* (MW539687) forms a paraphyletic group with *C. zawadskii* subsp. *coreanum* and *C. zawadskii* (MG799556), ii) *C. zawadskii* var. *latilobum* (MF034026) is placed separately from two *C. zawadskii* and *C. zawadskii* subsp. *coreanum*, and iii) *C. zawadskii*, *C. indicum*, and *C. boreale* do not form a monophyletic group (Figure 1), which is congruent to the previous phylogenetic study using chloroplast and nuclear regions (Liu et al. 2012). Our results present that the multiple times of evolutionary events, such as hybridization and introgression, have been occurred in *Chrysanthemum* genus once morphological classification is enough clear.

## Data Availability

Chloroplast genome sequence can be accessed via accession number of MW539687 in GenBank of NCBI at https://www.ncbi.nlm.nih.gov. The associated BioProject, SRA, and Bio-Sample numbers are PRJNA688416, SAMN17175002, and SRR13320595, respectively.
